# Hypothalamic administration of sargahydroquinoic acid elevates peripheral thermogenic signaling and ameliorates high fat diet-induced obesity through the sympathetic nervous system

**DOI:** 10.1038/s41598-021-00074-3

**Published:** 2021-10-29

**Authors:** Doyeon Kim, Yuna Lee, Hyeung-Rak Kim, Yeo Jin Park, Hongik Hwang, Hyewhon Rhim, Taek Kang, Chun Whan Choi, Bonggi Lee, Min Soo Kim

**Affiliations:** 1 Brain Science Institute, Korea Institute of Science and Technology (KIST), Seoul, 02792 Republic of Korea; 2 Division of Bio-Medical Science and Technology, KIST School, University of Science and Technology, Seoul, 02792 Republic of Korea; 3 Department of Food Science and Nutrition, Pukyong National University, Busan, 48513 Republic of Korea; 4 Korea Medicine (KM) Application Center, Korea Institute of Oriental Medicine, Daegu, 41062 Republic of Korea; 5 Korean Convergence Medicine, University of Science and Technology, Daejeon, 34504 Republic of Korea; 6 Natural Product Research Team, Gyeonggi Biocenter, Gyeonggido Business and Science Accelerator, Suwon, Gyeonggi-Do 16229 Republic of Korea

**Keywords:** Drug discovery, Molecular biology, Physiology, Endocrinology

## Abstract

*Sargassum serratifolium* (C. Agardh) C.Agardh, a marine brown alga, has been consumed as a food and traditional medicine in Asia. A previous study showed that the meroterpenoid-rich fraction of an ethanolic extract of *S. serratifolium* (MES) induced adipose tissue browning and suppressed diet-induced obesity and metabolic syndrome when orally supplemented. Sargahydroquinoic acid (SHQA) is a major component of MES. However, it is unclear whether SHQA regulates energy homeostasis through the central nervous system. To examine this, SHQA was administrated through the third ventricle in the hypothalamus in high-fat diet-fed C57BL/6 mice and investigated its effects on energy homeostasis. Chronic administration of SHQA into the brain reduced body weight without a change in food intake and improved metabolic syndrome-related phenotypes. Cold experiments and biochemical analyses indicated that SHQA elevated thermogenic signaling pathways, as evidenced by an increase in body temperature and UCP1 signaling in white and brown adipose tissues. Peripheral denervation experiments using 6-OHDA indicated that the SHQA-induced anti-obesity effect is mediated by the activation of the sympathetic nervous system, possibly by regulating genes associated with sympathetic outflow and GABA signaling pathways. In conclusion, hypothalamic injection of SHQA elevates peripheral thermogenic signaling and ameliorates diet-induced obesity.

## Introduction

Obesity is a global pandemic that arises from an imbalance between energy intake and expenditure. Because obesity is generally related to a variety of metabolic disorders such as cardiovascular diseases, diabetes, hypertension, and fatty liver, numerous attempts have been made to fight obesity^1^. Although various anti-obesity approaches can be chosen, pharmacotherapy is a popular way to ameliorate severe obesity^2^. Orlistat is probably the only anti-obesity drug approved for long-term use, but its effect is mild, and unfavorable side effects have been reported^3^. In addition, many anti-obesity drugs have been withdrawn from the market because of serious side effects^2^. Therefore, natural products and their derivatives have attracted attention for developing safer therapeutics for combating obesity.

Brown adipose tissue (BAT) is the main organ that maintains body temperature by dissipating energy as heat. Similarly, the conversion of white adipocytes to beige adipocytes, called the browning of white adipose tissue (WAT), has also been highlighted as a strategy to reduce obesity by elevating thermogenesis. BAT activation and adipocyte browning are induced by the direct effects of peripheral factors on adipocytes, such as thyroid prohormone thyroxine (T4), retinoblastoma interacting zinc finger protein homology domain containing 16 (PRDM16), chronic cold exposure, exercise, peroxisome proliferator-activated receptor γ (PPARγ) agonists, β3-adrenergic receptors, irisin, and norepinephrine^4, 5^. Thermogenesis can also be regulated by the central nervous system (CNS). The hypothalamus can regulate outflow signaling that drives sympathetic nerve activity to WAT and BAT, regulating energy homeostasis and heat generation^6^. Various signals associated with energy homeostasis are sent to the hypothalamus and integrated to affect expression of orexigenic or anorexigenic peptides that control feeding behavior. In addition, the hypothalamus can regulate glucose and lipid metabolism in peripheral tissues via the autonomic nervous system, including WAT browning and BAT activation^6–8^. Growing evidence indicates that WAT browning and BAT activation are attractive mechanisms for pharmaceutical intervention to obtain anti-obesity and anti-metabolic syndrome effects by elevating energy expenditure^5, 9, 10^.

*Sargassum serratifolium* (C.Agardh) C.Agardh, a marine brown alga belonging to the Sargassaceae family, mainly inhabits the coastal areas of Korea and Japan. Previous studies have shown that *S. serratifolium* contains high levels of meroterpenoids, including sargahydroquinoic acid (SHQA), sargachromenol, and sargaquinoic acid, of which SHQA is the most abundant compound^11–14^. A previous study reported that the meroterpenoid-rich fraction of an ethanolic extract of S. serratifolium (MES) inhibited diet-induced obesity and related metabolic syndrome^14^. Oral MES supplementation notably decreased body weight, fatty liver, and improved blood lipid profile without altering food intake in high-fat diet (HFD)-fed mice. Especially in the adipose tissue of mice with MES supplementation, uncoupling protein 1 (UCP1)-positive cells and related signaling were significantly elevated while macrophage infiltration was reduced^14^. Another study examined the direct effect of SHQA on adipocyte metabolism^15^. SHQA appears to activate lipid catabolic and adipocyte browning pathways, presumably by activating PPARγ, PPARα, and AMPKα signaling^15^. Although MES supplementation was effective for weight loss in mice and direct treatment of adipocytes with SHQA elevated signaling pathways related to lipid catabolism^14, 15^, it is unclear whether SHQA regulates energy homeostasis through the CNS. In this study, SHQA was administered through a cannula into the third ventricle (3V) of the brain and its effect on energy homeostasis in HFD-fed mice was investigated.

## Results

### Acute treatment with SHQA increases the neuronal excitability of hypothalamic ARC neurons

**Figure 1:**
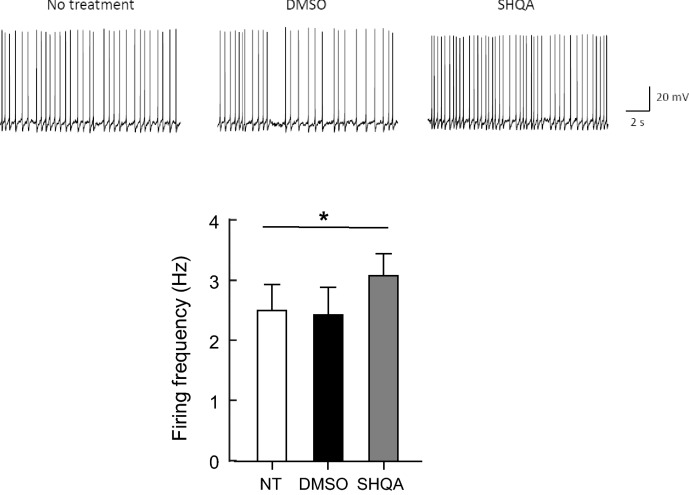
SHQA increases the frequency of spontaneous firing in ARC neurons. Hypothalamic ARC neurons were whole-cell patched under a current-clamp configuration and a change in the spontaneous firing frequency was monitored before and after the application of DMSO or SHQA (4.3 ng/µl). Data are expressed as mean ± S.E.M. (n = 5). Statistical differences between no treatment (NT), DMSO, and SHQA were determined using one-way ANOVA followed by Tukey’s post hoc test: *p < 0.05.

We determined whether acute treatment with SHQA affected the intrinsic neuronal excitability of hypothalamic ARC neurons. To this end, ARC neurons on acutely prepared hypothalamic brain slices were whole-cell patched under a current-clamp configuration, and changes in the firing frequency were monitored. We found that ARC neurons continuously fired action potentials at high frequencies even in the absence of any external stimulation (2.55 ± 0.42 Hz) and that treatment with DMSO vehicle did not affect the frequency of spontaneous firing (2.47 ± 0.45 Hz). However, when the hypothalamic slices were acutely treated with SHQA (4.3 ng/μL), the frequency of spontaneous firing was increased to 3.12 ± 0.36 Hz (Fig. 1), indicating that SHQA increases the intrinsic neuronal excitability of hypothalamic ARC neurons.

### Administration of SHQA into the 3V decreased HFD-induced obesity

**Figure 2:**
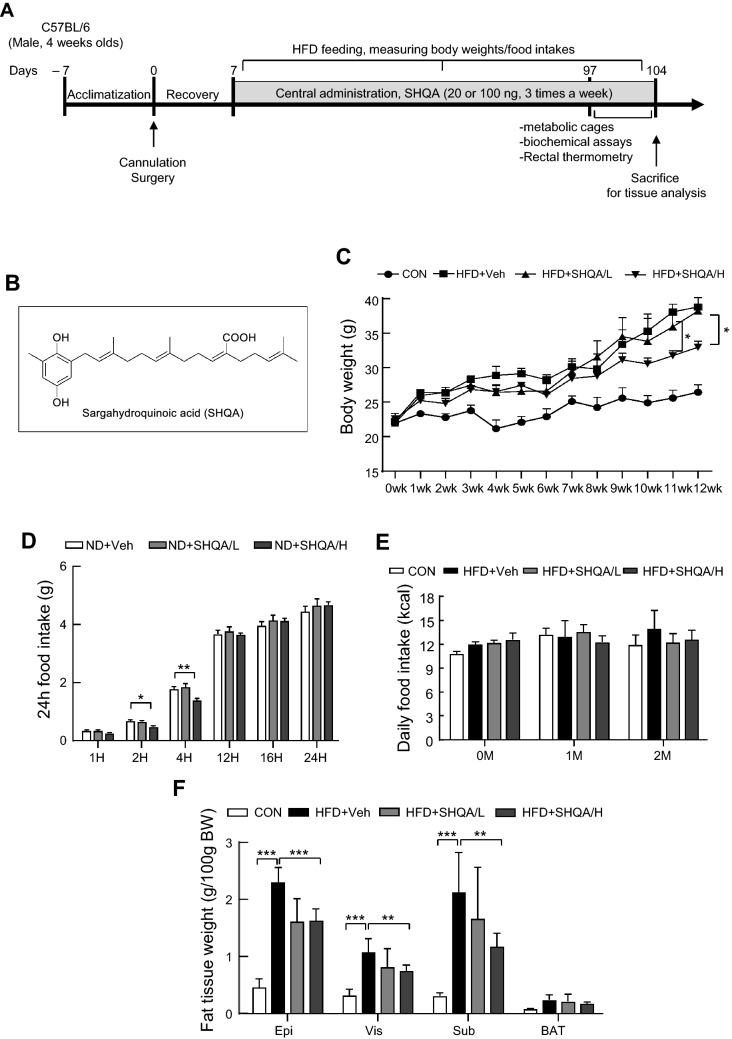
Hypothalamic SHQA injection ameliorates HFD-induced obesity. (**A**) The schematic timeline of the experimental schedule. (**B**) The chemical structure of SHQA. C57BL/6 mice (4 weeks old) were fed a chow diet (CON) or a high-fat diet (HFD) with the hypothalamic injection of PBS (CON or HFD + Veh, three times a week), a low dose (HFD + SHQA/L, 20 ng/µl, three times a week) or a high dose of SHQA (HFD + SHQA/H, 100 ng/µl, three times a week) for 12 weeks. (**C**) Body weight was measured every week. (**D**) Accumulated 24 h—food intake of normal diets (Chow, ND) and (**E**) chronic (2 months) food intake were measured. (**F**) Adipose tissue weight was measured at the end of the experiment. Data are expressed as mean ± S.E.M. (n = 6–7). Statistical differences between CON, HFD + Veh, HFD + SHQA/L (SHQA, 20 ng/µl), and HFD + SHQA/H (SHQA, 100 ng/µl) groups were determined using one-way ANOVA followed by the Tukey’s post hoc test: *p < 0.05, **p < 0.01, ***p < 0.001.

SHQA is a major component in the meroterpenoid-rich fraction of an ethanolic extract of Sargassum serratifolium and its structure is presented in Fig. 2B. SHQA is an isoprenoid quinone also found in other species of Sargassum^16–20^; it possesses an isoprenoid chain and a hydroquinone moiety. Based on the beneficial effects of SHQA on neuronal excitability, we further examined whether hypothalamic SHQA injection regulates energy balance (Fig. 2A). To examine whether central administration of SHQA is effective in regulating energy balance, C57BL/6 mice were fed a HFD (60% of calories as fat) with 3V injection of SHQA at low and high doses (20 ng/µl and 100 ng/µl, respectively) and body weight and food intake were monitored. As expected, HFD notably increased body weight, but SHQA administration, especially at a high dose, significantly inhibited body weight gain (Fig. 2C). Consistently, SHQA decreased the weights of fat depots at different sites (Fig. 2F), indicating that central administration of SHQA is effective in reducing HFD-induced weight gain. To examine whether the SHQA-mediated change in energy balance is due to an alteration in feeding behavior, we measured food intake at various time points under normal diet conditions (ND-fed). Although high doses of SHQA slightly reduced food intake at 2 h and 4 h, no apparent changes were observed after 24 h (Fig. 2D). Consistently, food intake (7 d) was unaltered among groups at 1 and 2 months of age (Fig. 2E), suggesting that SHQA did not significantly affect food intake in the CNS.

### Administration of SHQA into the 3V controls energy expenditure

**Figure 3:**
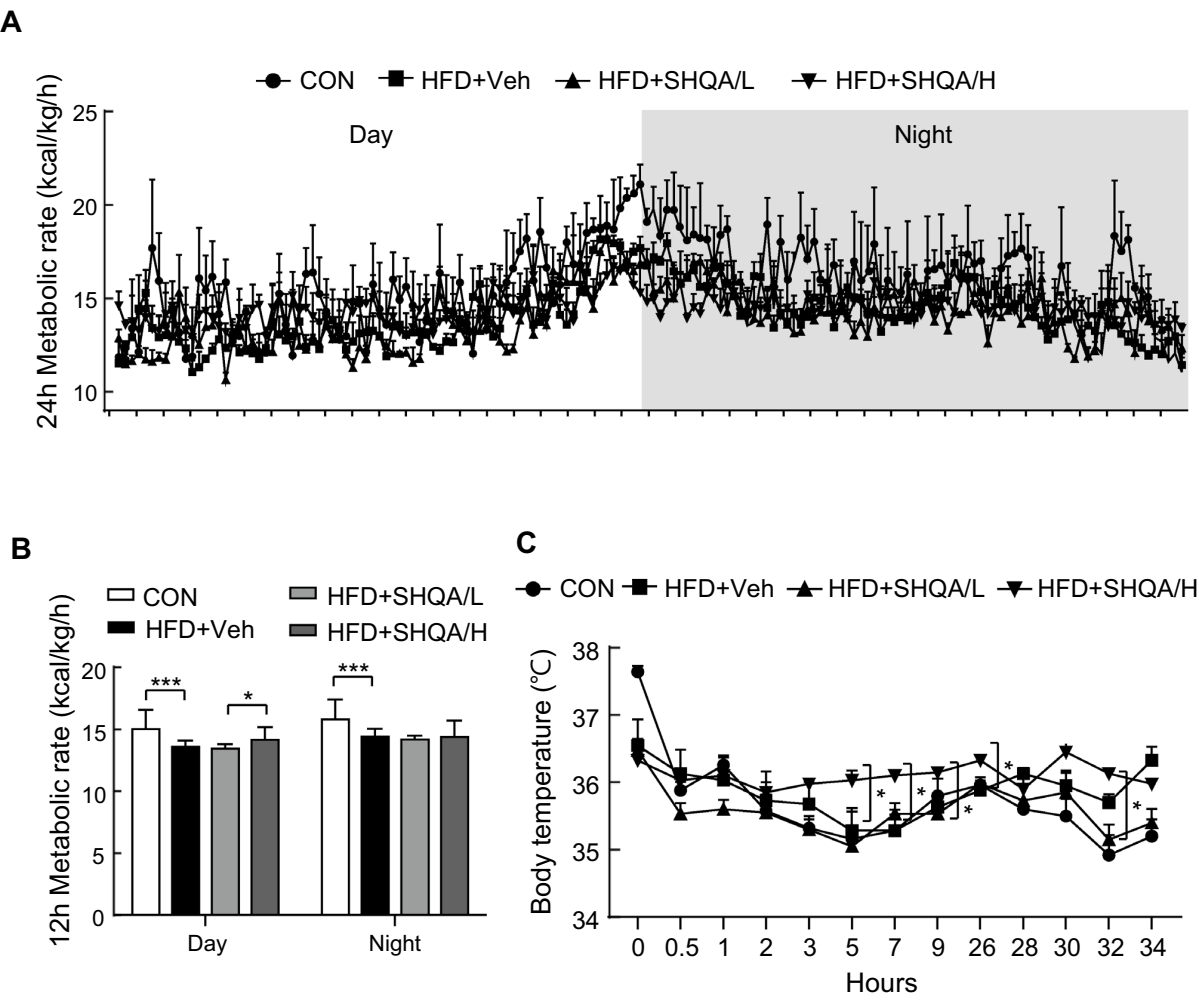
Hypothalamic injection of SHQA elevates energy expenditure and thermogenesis. C57BL/6 mice (4 weeks old) were fed a chow diet (CON) or a HFD with the hypothalamic injection of PBS (CON or HFD + Veh, three times a week), a low dose (HFD + SHQA/L, 20 ng/µl, three times a week) or a high dose of SHQA (HFD + SHQA/H, 100 ng/µl, three times a week) for 12 weeks. Indirect calorimetry was applied to measure the energy expenditure of the mice fed a chow diet or HFD with or without SHQA injection during the light and dark cycle. (**A**) 24 h metabolic rate (kcal/kg/h) was automatically calculated by the machine based on oxygen consumption and carbon dioxide production of mice. (**B**) The values of the metabolic rate were averaged and converted to the bar graphs. (**C**) To investigate adaptive thermogenesis, a rectal thermometer was applied to mice fed a chow diet or HFD with or without SHQA injection and the temperature was recorded in a cold room at 4 °C for 34 h. Data are expressed as mean ± S.E.M. (n = 6–7). Statistical differences between CON, HFD + Veh, HFD + SHQA/L (SHQA, 20 ng/µl), and HFD + SHQA/H (SHQA, 100 ng/µl) were determined using one-way ANOVA followed by the Tukey’s post hoc test: *p < 0.05, ***p < 0.001.

Because SHQA decreased body weight without altering food intake, we hypothesized that hypothalamic SHQA may elevate energy expenditure. To determine this, indirect calorimetry was performed during the light and dark cycles using mice fed a chow diet or HFD with or without SHQA injection. The data showed that SHQA injection at a high concentration elevated the metabolic rate during the light cycle (Fig. 3A,B). To further test whether the increase in metabolic rate in the SHQA-injected group was associated with elevation of thermogenic energy expenditure, a cold challenge test was performed. The data showed that the rectal body temperature was higher in mice injected with a high concentration of SHQA than HFD-fed control mice maintained in a cold chamber (4 °C) (Fig. 3C), indicating that hypothalamic SHQA-mediated thermogenesis may contribute to the increase in energy expenditure.

### Hypothalamic administration of SHQA ameliorates blood triglyceride levels after HFD

**Figure 4:**
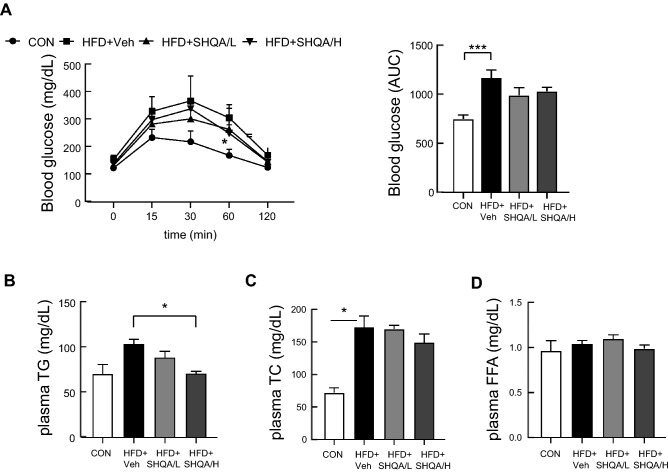
Hypothalamic injection of SHQA improves the metabolic profile in HFD-fed mice. Standard C57BL/6 mice (4 weeks old) were fed a chow diet (CON) or a HFD with the hypothalamic injection of PBS (CON or HFD + Veh, three times a week), a low dose (HFD + SHQA/L, 20 ng/µl, three times a week) or a high dose of SHQA (HFD + SHQA/H, 100 ng/µl, three times a week) for 12 weeks. The blood of these mice was collected for measuring metabolic profiles. (**A**) A glucose tolerance test was performed using 12 wk-old mice fed a chow diet or HFD with or without SHQA injection. The area under the curve was calculated using the values from (**A**). (**B**) Total triglycerides, (**C**) Total cholesterols, and (**D**) Free fatty acids in the blood were determined using commercially available kits. Data are expressed as mean ± S.E.M. (n = 6–7). Statistical differences between CON, HFD + Veh, HFD + SHQA/L (SHQA, 20 ng/µl), and HFD + SHQA/H (SHQA, 100 ng/µl) were determined using one-way ANOVA followed by the Tukey’s post hoc test: *p < 0.05, ***p < 0.001.

We examined whether SHQA-induced body weight reduction is related to the improvement of metabolic parameters by measuring blood glucose and lipid profiles. The glucose tolerance test showed that the HFD-induced increase in blood glucose levels was not affected by SHQA injection (Fig. 4A). Despite no clear effects of SHQA on free fatty acids (FFAs) and total cholesterol levels in the blood, the levels of triglycerides were reduced by SHQA injection (Fig. 4B–D). We hypothesize that the body weight-lowering effect of hypothalamic SHQA injection is moderate and may not be sufficient to recover the blood metabolic profile except for triglycerides.

### Hypothalamic administration of SHQA upregulates mRNA levels of thermogenic genes in BAT

**Figure 5:**
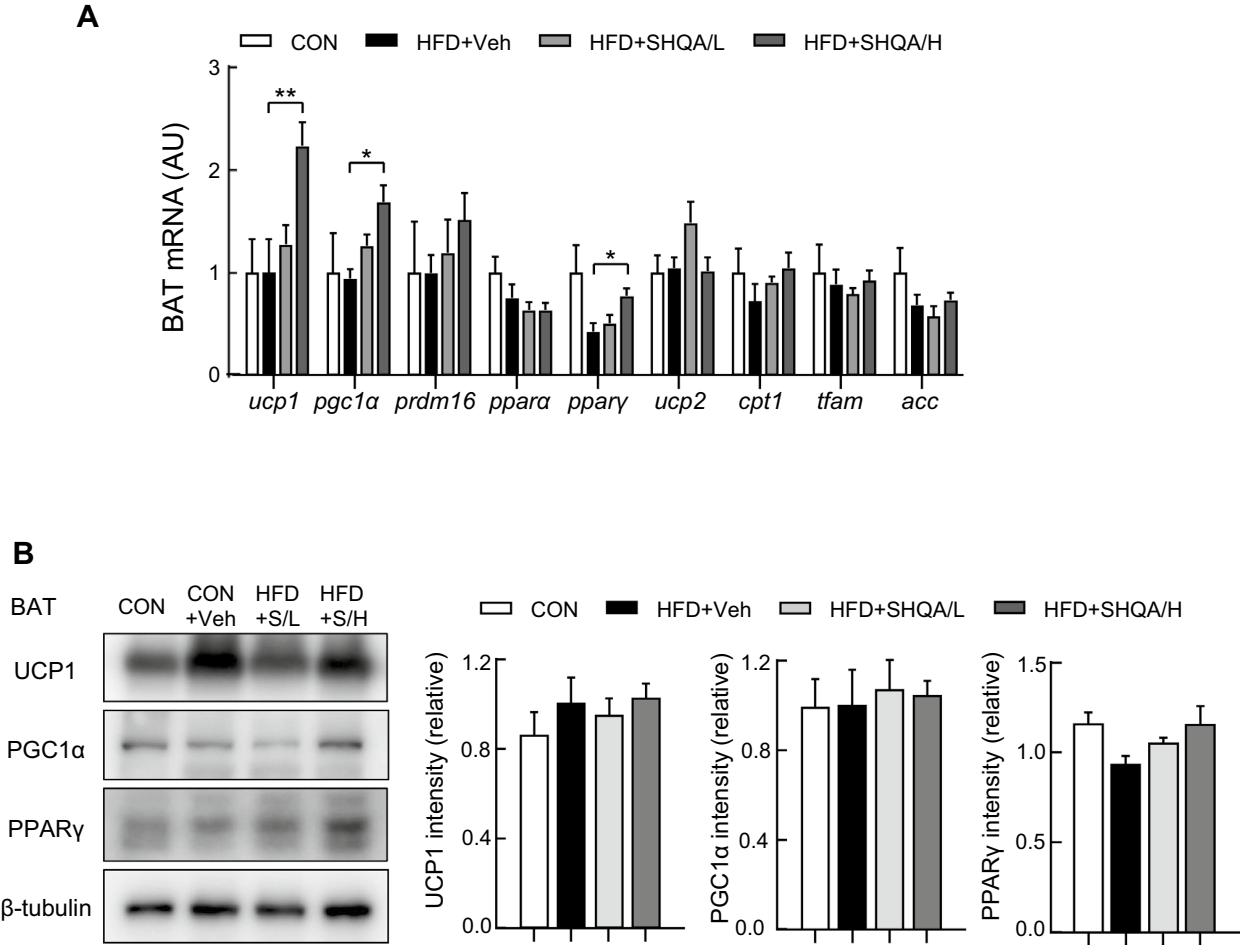
SHQA injection alters gene expression signature in brown adipose tissues of mice. C57BL/6 mice (4 weeks old) were fed a chow diet (CON) or a HFD with the hypothalamic injection of PBS (CON or HFD + Veh, three times a week), a low dose (HFD + SHQA/L or HFD + S/L, 20 ng/µl, three times a week) or a high dose of SHQA (HFD + SHQA/H or HFD + S/H, 100 ng/µl, three times a week) for 12 weeks. The brown adipose tissues from these mice were collected for measuring mRNA and protein levels. (**A**) mRNA expression levels of genes related to adipose tissue browning and thermogenesis were measured in brown adipose tissues (n = 6-7). (**B**) The protein levels of UCP1, PGC1α, and PPARγ in BAT were measured by western blots (n = 4-5) with only representative bands shown in the figure (whole images of blot were shown in Supplementary Fig. S1). Data are expressed as mean ± S.E.M. Statistical differences between CON, HFD + Veh, HFD + SHQA/L (SHQA, 20 ng/µl), and HFD + SHQA/H (SHQA, 100 ng/µl) were determined using one-way ANOVA followed by the Tukey’s post hoc test (**A** and **B**): *p < 0.05, **p < 0.01 compared to HFD+Veh.

Oral MES supplementation improved diet-induced obesity and metabolic syndrome partially by elevating signals associated with lipid catabolism, including lipolysis, mitochondrial biogenesis, and the number of UCP1-positive cells in adipose tissues^14^. We investigated whether hypothalamic injection of SHQA can control genes related to lipid catabolism, including thermogenic signaling pathways in BAT using qPCR. In BAT of chow and HFD-fed mice, no significant changes were observed in the mRNA levels of genes associated with BAT activation and functions such as UCP1, UCP2, peroxisome proliferator-activated receptor gamma co-activator 1 (PGC1α), and PR-domain containing 16 (PRDM16), lipid catabolism, such as carnitine palmitoyltransferase I (CPT1), mitochondrial transcription factor A (TFAM), acetyl-CoA carboxylase (ACC), and lipid metabolism-related transcription factors, including peroxisome proliferator-activated receptor α (PPARα) and PPARγ (Fig. 5A). Although SHQA injection at a low concentration did not show significant effects, the high concentration significantly upregulated the mRNA levels of UCP1, PGC1α, and PPARγ in BAT compared to the HFD-fed control group (Fig. 5A). However, no dramatic changes were observed in the protein levels of UCP1, PGC1α, and PPARγ (Fig. 5B), indicating that the thermogenic effect of hypothalamic SHQA may not be due to BAT-mediated thermogenesis.

### Increased thermogenesis signaling in WAT by central administration of SHQA

**Figure 6:**
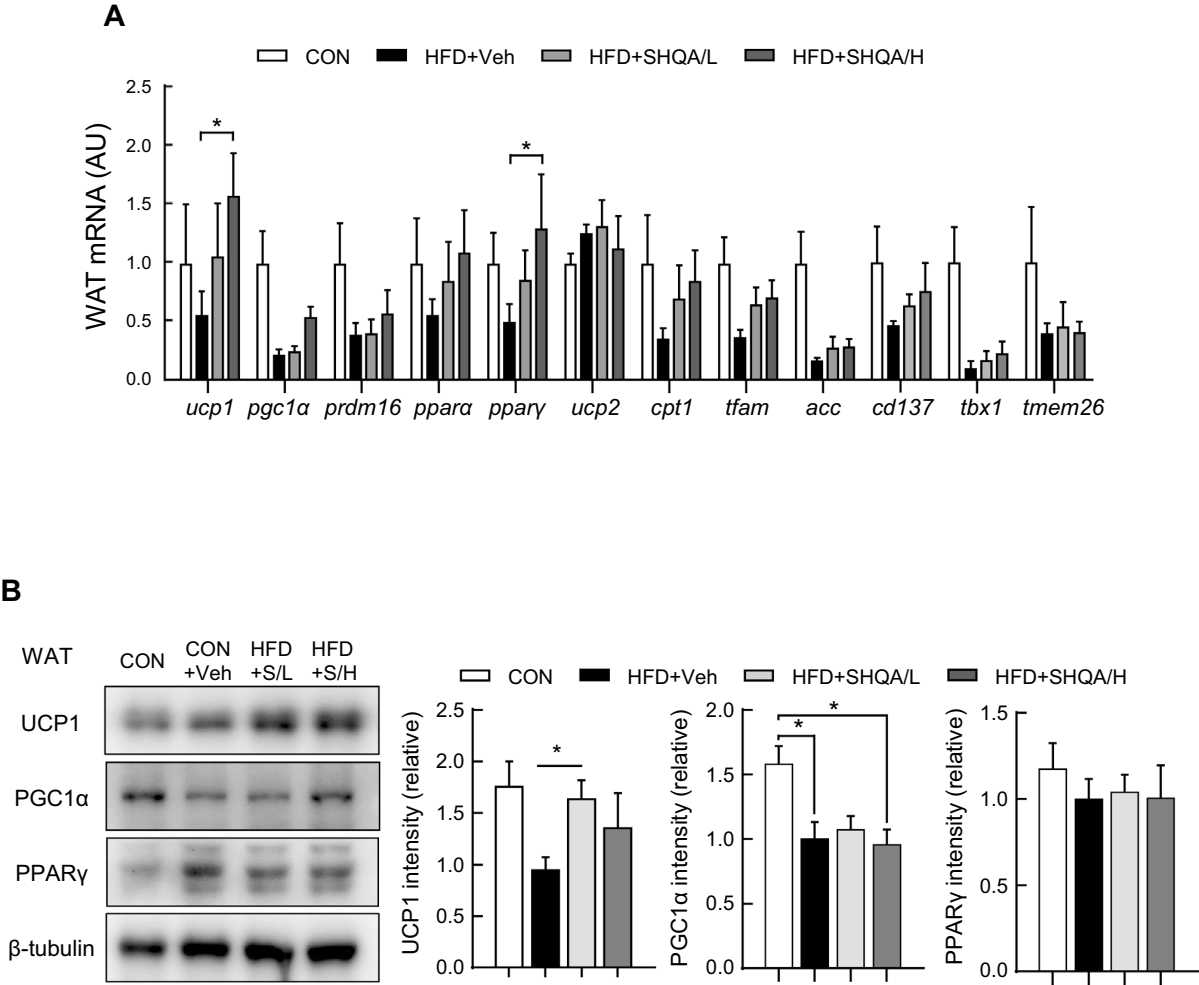
SHQA injection alters gene expression signature in white adipose tissues of mice. C57BL/6 mice (4 weeks old) were fed a chow diet (CON) or a HFD with the hypothalamic injection of PBS (CON or HFD + Veh, three times a week), a low dose (HFD + SHQA/L or HFD + S/L, 20 ng/µl, three times a week) or a high dose of SHQA (HFD + SHQA/H or HFD + S/H, 100 ng/µl, three times a week) for 12 weeks. The subcutaneous white adipose tissues from these mice were collected for measuring mRNA and protein levels. (**A**) mRNA expression levels of genes related to adipose tissue browning and thermogenesis were measured in white adipose tissues. (**B**) The protein levels of UCP1, PGC1α, and PPARγ were measured by western blots with representative bands shown in the figure (whole images of blot were shown in Supplementary Fig. S2). Data are expressed as mean ± S.E.M. Statistical differences between CON, HFD + Veh, HFD + SHQA/L (SHQA, 20 ng/µl), and HFD + SHQA/H (SHQA, 100 ng/µl) were determined using one-way ANOVA followed by the Tukey’s post hoc test: *p < 0.05; Statistical differences between HFD + Veh and HFD + SHQA/L in UCP1 protein were determined using two-tailed student’s *t* test: *p < 0.05; n = 6–7 mice for qPCR (**A**) and n = 4–5 for western blotting (**B**).

We also investigated whether hypothalamic injection of SHQA could control genes related to lipid catabolism, including thermogenic signaling pathways in WAT. Of the various genes examined in BAT, the mRNA levels of UCP1 and PPARγ were significantly increased in WAT by SHQA treatment (Fig. 6A). However, no significant changes were observed in the mRNA levels of PGC1α, PRDM16, PPARα, UCP2, CPT1, TFAM, and ACC following hypothalamic SHQA injection (Fig. 6A). Consistently, SHQA treatment increased UCP1 protein levels (Fig. 6B), suggesting that SHQA-induced thermogenic signaling in WAT contributes to its anti-obesity effect.

### SHQA broadly alters the mRNA expression levels of genes related to sympathetic outflow and GABA signaling pathways

**Figure 7:**
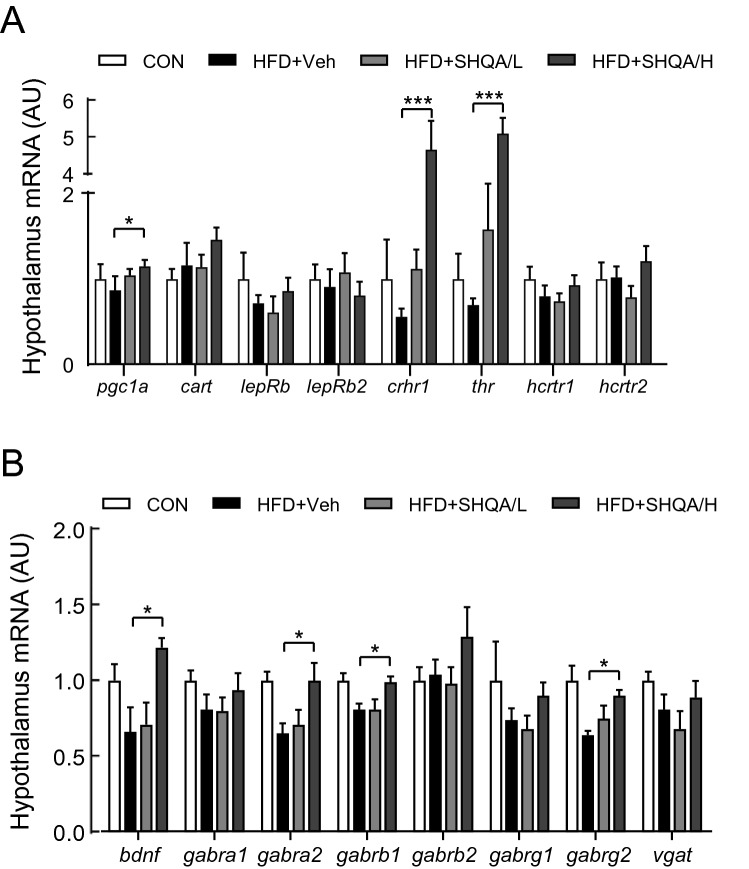
SHQA injection alters gene expression profile in the hypothalamus. C57BL/6 mice (4 weeks old) were fed a chow diet (CON) or a HFD with the hypothalamic injection of PBS (CON or HFD + Veh, three times a week), a low dose (HFD + SHQA/L, 20 ng/µl, three times a week) or a high dose of SHQA (HFD + SHQA/H, 100 ng/µl, three times a week) for 12 weeks. The hypothalamus from these mice was collected for measurements of mRNA levels. mRNA expression levels of genes related to (**A**) sympathetic outflow and (**B**) GABAnergic signaling pathways were measured in the hypothalamus of 12 wk-old mice fed a chow diet or HFD with or without SHQA injection. Data are expressed as mean ± S.E.M. (n = 6–7). Statistical differences between CON, HFD + Veh, HFD + SHQA/L, and HFD + SHQA/H were determined using one-way ANOVA followed by Tukey’s post hoc test: *p < 0.05, ***p < 0.001.

The hypothalamus is the primary brain region that integrates the outflow signals that induces sympathetic nerve activity to BAT and WAT, stimulating thermogenesis, and regulating energy homeostasis. To investigate the potential mechanisms underlying the hypothalamic SHQA injection-mediated stimulation of thermogenesis, we measured mRNA expression levels of several genes associated with sympathetic nerve activity and thermogenesis in the hypothalamus, including CART, LepRb, LepRb2, PGC1α, CRHR1, THR, HCRTR1, HCRT2, BDNF, GABRA1, GABRA2, GABRB1, GABRB2, GABRG1, GABRG2, and VGAT. Of these genes, the mRNA levels of PGC1α were slightly increased and those of CRHR1 and THR were most notably increased by a hypothalamic SHQA injection at a high concentration (Fig. 7A), indicating that SHQA may stimulate neural signaling pathways involved in energy homeostasis mediated by corticotropin-releasing factor and thyroid hormone. SHQA also significantly upregulated the mRNA levels of BDNF, GABRA2, GABRB1, and GABRG2 in the hypothalamus (Fig. 7B). Because BDNF has been shown to boost sympathetic outflow and GABAergic signaling acting on the paraventricular nucleus of the hypothalamus and induce thermogenesis, SHQA-stimulated BDNF upregulation and GABA-related signaling may contribute to the elevation of peripheral thermogenic signaling.

### The brain-fat tissue denervation disrupted the body weight-lowering effect of SHQA

**Figure 8:**
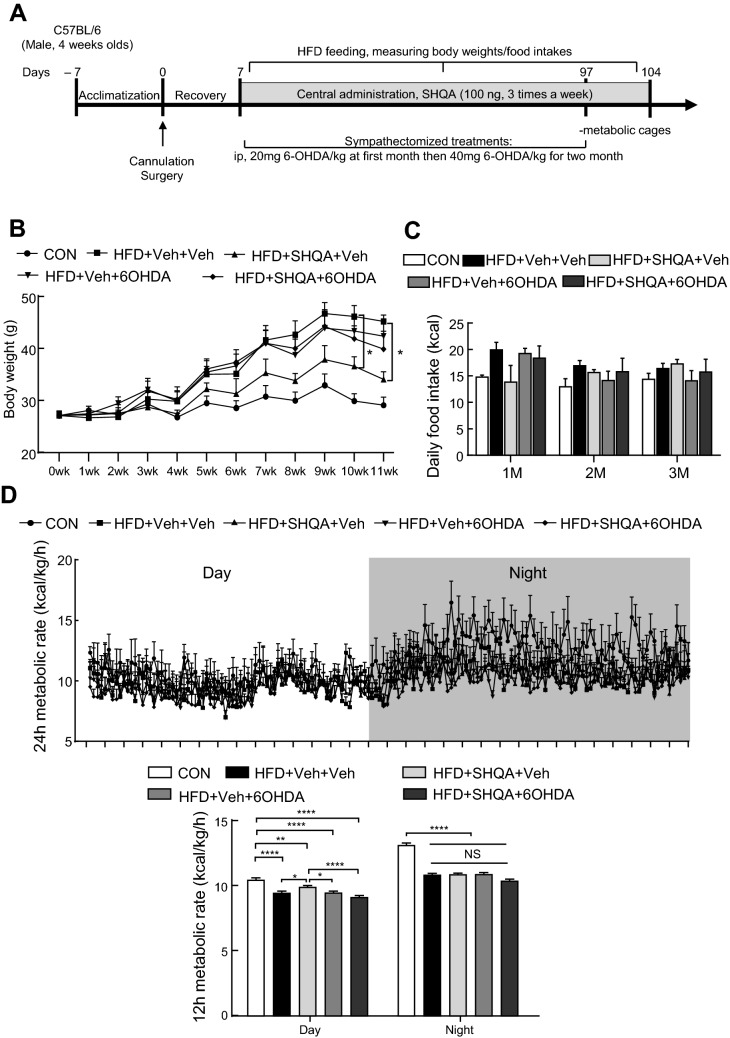
Hypothalamic injection of SHQA induces metabolic regulation by brain-fat tissue innervation. C57BL/6 mice were fed and treated with CON (chow diets), HFD + Veh + Veh (60% of calorie as fat, central injection with PBS, intraperitoneal injection with PBS), HFD + SHQA + Veh (60% of calorie as fat, central injection with 100 ng/µl SHQA, intraperitoneal injection with PBS), HFD + Veh + 6OHDA (60% of calorie as fat, central injection with PBS, intraperitoneal injection with 6-OHDA) or HFD + SHQA + 6OHDA (60% of calorie as fat, central injection with 100 ng/µl SHQA, intraperitoneal injection with 6-OHDA) groups. (**A**) The schematic timeline of the experimental schedule. (**B**) Weekly body weight was measured for 11 weeks. (**C**) Monthly food intakes were recorded. (**D**) At 12 weeks, indirect calorimetry was applied to measure the energy expenditure of these mice, and 24-h metabolic rates (kcal/kg/h) were automatically calculated by the machine based on oxygen consumption and carbon dioxide production of mice. The values of the metabolic rate were averaged and converted to the bar graphs. Data are expressed as mean ± S.E.M. Statistical differences between CON, HFD + Veh, HFD + SHQA (SHQA, 100 ng/µl), HFD + 6OHDA + Veh and HFD + 6OHDA + SHQA groups were determined using one-way ANOVA followed by the Tukey’s post hoc test: *p < 0.05, **p < 0.01, ****p < 0.0001; n = 3–5 mice for weekly body weight (**B**), n = 3–5 mice for monthly food intake (**C**) and n = 4–5 mice for metabolic analysis (**D**).

The schematic timeline of the experimental schedule is shown in Fig. 8A. It is known that thermogenesis in brown and beige adipose tissues is stimulated by the activation of the sympathetic nervous system in the hypothalamus^6, 21, 22^. Based on the data showing that hypothalamic injection of SHQA elevated metabolic rate and thermogenic signaling, we hypothesized that SHQA regulates the hypothalamus-adipose tissue axis through the sympathetic nervous system and investigated whether brain-fat innervation induces thermogenic signaling by the IP injection of 6-hydroxydopamine (6-OHDA), a chemical compound for sympathetic denervation in experimental mice^23, 24^. To examine whether peripheral denervation by 6-OHDA alters the SHQA-mediated beneficial effects on energy balance, C57BL/6 mice were fed and treated with CON (chow diets with central injection of PBS and intraperitoneal injection of PBS), HFD + Veh + Veh (fed HFD with central injection of PBS and intraperitoneal injection of PBS), HFD + SHQA + Veh (fed HFD with central injection of 100 ng/µl SHQA and intraperitoneal injection of PBS), HFD + Veh + 6OHDA (fed HFD with central injection of PBS and intraperitoneal injection of 6-OHDA), and HFD + SHQA + 6OHDA (fed HFD with central injection of 100 ng/µl SHQA and intraperitoneal injection of 6-OHDA). As expected, the HFD + Veh + Veh group had notably increased body weight, which was partially reversed by hypothalamic SHQA injection (Fig. 8B). 6-OHDA injection in HFD-fed mice notably increased body weight, indicating that 6-OHDA injection itself did not interrupt HFD-induced body weight gain (Fig. 8B). While hypothalamic SHQA injection significantly reduced body weight, SHQA injection in mice with peripheral denervation did not alter body weight (Fig. 8B). In addition, 6-OHDA injection or 6-OHDA injection with central SHQA administration did not affect food intake (Fig. 8C). We further examined whether SHQA-mediated elevation of energy expenditure was due to the sympathetic nervous system by indirect calorimetry. The data showed that the SHQA injection (a high concentration) elevated the metabolic rate during the light cycle, but no dramatic change was found in the denervated mice treated with SHQA during the night cycle (Fig. 8D). These data indicate that hypothalamic SHQA alters energy balance through the sympathetic nervous system.

## Discussion

Our previous study showed that MES induced thermogenic signaling in adipose tissues and improved diet-induced obesity and metabolic syndrome when supplemented with a HFD^14^. The chemical composition analysis suggested that SHQA is the primary compound in MES^14^. Further in vitro studies indicated that SHQA treatment elevated mitochondrial numbers and thermogenic signaling pathways in adipocytes. However, it is questionable whether SHQA functions in the central nervous system to regulate energy balance. Based on the current study, it is clear that hypothalamic injection (3 V) of SHQA significantly improved HFD-induced body weight gain and metabolic profile partly through the increase in thermogenic signaling in adipose tissues. The underlying mechanisms include, but are not limited to, the broad up-regulation of genes associated with sympathetic outflow and GABA-related signaling pathways.

It has been reported that short-term HFD feeding (2–4 weeks) changes expression levels of proteins, including UCP1, CPT2, ACOT1, and ACOT2 in BAT. However, in BAT of mice fed HFD for the long term, these protein levels were nearly the same as those in BAT of the chow-fed control group^25^. The study suggested that lipid catabolism pathways are elevated in BAT in response to HFD, but severe obesity caused by prolonged HFD feeding may deprive the homeostatic response of BAT^25^. Our results also support this conclusion because 12 weeks of HFD feeding did not notably change the mRNA levels of genes associated with lipid catabolic pathways, including UCP1, UCP2, and CPT1. We also hypothesize that profound obesity accompanied by dysregulation of lipid and glucose profiles in circulation may fade the adaptive response to HFD, although the precise mechanisms need to be further elucidated.

The proper cold-exposure conditions that induce adipose tissue browning are diverse and depend on the experimental conditions^26–29^. A big data analysis using GeneChip array^26^ and RNA-seq analyses^29^ showed that 4 or 24-h cold exposure dramatically changes the gene expression profile in WAT. Histological analysis of subcutaneous WAT of mice exposed to acute cold stimulation (4 °C for 4 h) revealed the appearance of beige adipocytes in subcutaneous WAT. RNA-seq analysis showed that 714 genes were differentially expressed, of which 221 genes were upregulated and 493 genes were downregulated^29^. Further gene ontology analyses indicated that the upregulated genes were associated with fatty acid oxidation, lipid metabolic processes, and others^29^. On the other hand, downregulated genes were related to immune response, regulation of response to stimulus, defense response, etc.^29^. Another study using GeneChip array indicated that 24-h cold exposure notably changed the gene expression profile related to energy metabolism in subcutaneous WAT^26^. We exposed the mice to cold (4 °C for 34 h) based on our preliminary experiments indicating clear adipose browning characteristics in the gene expression profile (unpublished data). However, further studies in different cold-exposure conditions (acute vs. chronic) may be necessary to reveal more detailed effects of SHQA on lipid catabolic pathways in adipose tissues. Boosting sympathetic outflow is important for integrating signaling from the central nervous system and adipose thermogenesis^21^. Several factors in the hypothalamus have been reported to stimulate the activation of BAT and WAT browning. It has been reported that BDNF neurons in the medial and posterior paraventricular nucleus of the hypothalamus stimulate thermogenesis through the release of BDNF into the spinal cord to elevate sympathetic outflow^30^. In addition, corticotropin-releasing factors that act on hypothalamic corticotropin-releasing factor receptors are related to the regulation of energy balance. The central injection of corticotropin-releasing factor or microinjection of corticotropin-releasing factor into the preoptic area or the dorsomedial hypothalamus stimulated sympathetic nerve activity and temperature of BAT^31^. Thyroid hormones have also been shown to boost the sympathetic nervous system and stimulate thermogenesis in BAT and WAT browning partly by AMPK in the ventromedial nucleus of the hypothalamus^6^. Our study showed that SHQA significantly upregulated hypothalamic CRHR1, THR, and BDNF levels. Although the thermogenic mechanisms mediated by each gene may be different, these genes are closely associated with the stimulation of sympathetic outflow. In addition, the SHQA-mediated increase in energy expenditure and decrease in body weight of HFD-fed mice disappeared by sympathetic denervation using 6-OHDA, indicating that SHQA may stimulate sympathetic nerves in the hypothalamus for BAT activation and WAT browning, although more detailed experiments are necessary to elucidate the mechanism of this effect.

Hypothalamic GABA signaling is important for thermogenesis and energy expenditure^32^. Hypothalamic injections (the preoptic area) of GABA or a GABA receptor agonist elevated the rate of energy expenditure and body core temperature of urethane-chloralose-anesthetized, artificially ventilated rats^32^. In the same study, the author showed that GABA-mediated thermogenic energy expenditure was accompanied by a tachycardic response and electromyographic activity recorded from the femoral or neck muscles^32^. It appears that GABA signaling in the preoptic area of the hypothalamus mediates cold-induced thermogenesis. Hypothalamic injection of SHQA significantly upregulated the mRNA levels of GABRA2, GABRB1, and GABRG2 in the hypothalamus. We hypothesize that SHQA may also regulate hypothalamic GABA signaling to activate peripheral thermogenesis. It is necessary to further study whether SHQA controls tachycardic response and electromyographic activity related to GABA signaling pathways in the hypothalamus.

The role of AMPK in thermogenic energy expenditure differs between the hypothalamus and adipose tissue. A study using adipose tissue-specific AMPK knockout mice showed that AMPK is necessary for WAT browning and thermogenic energy expenditure^33^, whereas the activation of AMPK in the hypothalamus elevates food intake and reduces adaptive thermogenesis in adipose tissues to decrease energy expenditure in response to food deprivation^34^. Our previous study showed that SHQA treatment activates AMPK and lipid catabolic pathways, including thermogenic signaling in 3T3-L1 adipocytes^15^. In the current study, hypothalamic injection of SHQA in HFD-fed mice elevated peripheral thermogenic signaling and body temperature, thereby preventing diet-induced obesity. Although not directly tested here, we hypothesize that the effects of SHQA on hypothalamic AMPK may not be strong in reducing thermogenesis, unlike its effects on adipose tissues, or that other thermogenic signaling pathways in the hypothalamus induced by SHQA may be stronger than its effect on AMPK.

Although it is clear that high concentrations of SHQA (100 ng/µl) moderately elevate energy expenditure and decrease body weight in HFD-fed mice. Additionally, the cold challenge test showed that only a high concentration of SHQA elevated the body temperature compared to the HFD-fed control group. Thus, the thermogenic effects of SHQA were examined using qPCR and western blotting. However, there was a discrepancy between the mRNA and protein levels of genes associated with thermogenesis. qPCR data showed that only the high concentration of SHQA upregulated the mRNA levels of UCP1 in both WAT and BAT. However, both low and high concentrations of SHQA increased the protein levels of UCP1 only in WAT, without affecting those in BAT. These data indicate that UCP1 upregulation in WAT contributes to an increase in energy expenditure in the SHQA-injected group.

Despite the elevation of UCP1 protein levels in WAT, the mRNA levels of adipose browning markers, including CD137, TBX1, and TMEM26, were not different between the control and SHQA-injected groups. Nevertheless, UCP1 may be a functional marker of adipose browning. In support of this, treatment with isoproterenol, one of the most powerful browning agents, dramatically upregulated UCP1 in both white and beige adipocytes but did not alter the mRNA levels of CD137, TBX1, and TMEM26 in beige adipocytes and slightly upregulated these markers in white adipocytes, indicating that UCP1 protein is a more reliable marker for adipocyte browning^35^.

Our data demonstrated that high concentrations of SHQA elevated body temperature and reduced body weight in HFD-fed mice. These data raise a question about the relationship between upregulation of UCP1 and body weight reduction by SHQA treatment. Although both low (20 ng/µl) and high (100 ng/µl) concentrations of SHQA increased the protein levels of UCP1 in WAT, only the high concentration of SHQA elevated body temperature during the cold study and decreased body weight. These data indicate that UCP1 upregulation may not be the sole mechanism underlying SHQA-mediated thermogenesis and anti-obesity effects. Various studies support the existence of UCP1-independent thermogenic mechanisms^36^. Subcutaneous WAT of UCP1 KO mice housed in a chronic cold condition exhibited higher respiration than that of UCP1 KO mice housed in thermoneutrality^37^. In addition, chronic treatment with β3 adrenergic agonists elevated oxygen consumption in the WAT of UCP1 KO mice^38^. One of the mechanisms underlying UCP1-independent thermogenesis could be the creatine-substrate cycling^36, 39, 40^. Therefore, it is necessary to further study whether hypothalamic SHQA injection can regulate UCP1-independent thermogenesis and control energy expenditure regardless of thermogenesis, such as physical activity. It is also necessary to further examine the effects of hypothalamic injection of SHQA on metabolic changes in chow diet-fed control mice.

In conclusion, our data support that SHQA acts on the hypothalamus to induce peripheral thermogenesis and prevent HFD-induced obesity. The underlying mechanisms include but are not limited to SHQA-mediated alterations in the mRNA expression of genes associated with the sympathetic outflow and GABA signaling pathways. Thus, SHQA may be applied to pharmaceutical compounds that prevent obesity and obesity-related metabolic disorders.

## Methods

### Animals

Wild-type C57BL/6 male mice (4 weeks old, n = 27) were obtained from DBL Inc. (Saeron Bio Inc., Uiwang, Republic of Korea). The animals were housed in a room with a temperature of 23 ± 1 °C, a humidity of 50 ± 10%, a 12 h light/dark cycle, and free access to food and water. Following arrival, the mice were acclimatized to the Korea Institute of Science and Technology (KIST) animal room for one week. After one week of adaptation, the mice were randomly divided into four groups (n = 6–7). All mice underwent stereotaxic surgery and received the vehicle and experimental treatments for three months: (1) Control group (CON): mice fed chow diets and injected with the vehicle (PBS) with a guided cannula into the third ventricle, (2) HFD + Vehicle group (HFD + Veh): mice fed 60% HFD and injected with the vehicle (PBS) with a guided cannula into the third ventricle, (3) HFD + SHQA-low group (HFD + SHQA/L): mice fed a 60% HFD and injected with 20 ng/µl SHQA (low) with a guided cannula into third-ventricle, and (4) HFD + SHQA-high group (HFD + SHQA/H): mice fed 60% HFD and injected 100 ng/µl SHQA (high) with a guided cannula into the third-ventricle. The schematic timeline of the experimental schedule is shown in Fig. 2A. For chemically sympathectomized experiments, wild-type C57BL/6 male mice (4 weeks old) were subjected to the same stereotaxic surgery for cannulation in the third ventricle (3 V) of the hypothalamus, and then administered an intraperitoneal (i.p.) injection of 20 mg 6-OHDA (Sigma-Aldrich, St. Louis, MO, USA) /kg in PBS containing 0.1% ascorbic acid (Sigma, St. Louis, MO, USA) in the first month, followed by 40 mg 6-OHDA/kg or PBS for the following two months. These sympathectomy experiments were performed in mice fed with HFD for three months. Mice were divided into five groups (n = 4–5); (1) Control group (CON); mice fed chow diets, received vehicle via the 3V of the brain and intraperitoneally injected vehicle, (2) HFD + vehicle group (HFD + Veh + Veh): mice fed 60% HFD, received vehicle via the 3V of brain and intraperitoneally injected vehicle, (3) HFD + SHQA group (HFD + SHQA + Veh): mice fed 60% HFD, received SHQA (100 ng/µl) via 3V of brain intraperitoneally injected vehicle, (4) HFD + vehicle + 6-OHDA group (HFD + Veh + 6OHDA): mice fed 60% HFD, received vehicle via the 3V of the brain and intraperitoneally injected 6-OHDA, and (5) HFD + SHQA + 6-OHDA group (HFD + SHQA + 6OHDA): mice fed 60% HFD, received SHQA (100 ng/µl) via 3V of the brain and intraperitoneally injected 6-OHDA. The schematic timeline of the experimental schedule is shown in Fig. 8A. After we conducted live animal experiments (glucose tolerance test, body temperature experiments, and metabolic analysis), the tissues of these mice, including the hypothalamus, blood, brown adipose tissues, and subcutaneous white adipose tissues were aseptically collected by sacrificing the mice using a CO_2_ chamber. The Institutional Animal Care and Use Committee (IACUC) and the Institutional Biosafety Committee (IBC) at the KIST approved all procedures (Approval number KIST-2019–048). All experiments were performed in accordance with the relevant guidelines and regulations of the IACUC and IBC in KIST. The study was conducted in compliance with ARRIVE guidelines.

### Cannulation and chronic injection into the hypothalamic third ventricle

The hypothalamic third ventricle (3V) was cannulated as previously described^41, 42^. The ultraprecise small animal stereotactic apparatus (Kopf Instruments, Tujunga, CA, USA) was used to implant a 26-gauge guide cannula at the midline coordinates of 2.0 mm posterior to the bregma and 5.0 mm below the bregma. All mice underwent cannulation surgery independent of treatment. PBS, as a vehicle, and SHQA (20 or 100 ng/µl, three times a week) were injected via a cannula into the 3V of the brain for three months.

### Metabolic and energy expenditure analyses

To measure metabolic rates, metabolic parameters in the treated mice were analyzed with indirect calorimetry^43, 44^ using the Comprehensive Laboratory Animal Monitoring System (CLAMS) system (Columbus Instruments, Columbus, OH, USA) at normal temperature (22℃). The mice were individually housed 2–3 days before the experiment for habituation and then fed the same diet (chow or HFD) and water provided ad libitum in clear respiratory chambers (20.5 × 10.5 × 12.5 cm). O_2_ consumption, CO_2_ production, energy expenditure, heat, and activities of the experimental animals were measured for 24 h. These data were collected with Oxymax for Windows (version 5.40.14, Columbus Instruments) and analyzed with CLAX software (version 2.2.15, Columbus Instruments). Based on the collected data, the respiratory quotient or exchange ratio (CO_2_/O_2_) and delta-heat values were calculated using the CLAX software.

### Body temperature measurements

Rectal thermometry is a common method for measuring body temperature in mice^43, 45^. After central treatment of mice fed chow or HFD diet with vehicle (PBS), SHQA/L, and SHQA/H for three months, the mice were placed in a 4 °C cold room for two days. The decrease in body temperature of mice in the cold room was measured at 0, 0.5, 1, 2, 3, 5, 7, 9, 26, 28, 30, 32, and 34 h by inserting a small-diameter temperature probe > 2 cm into the anus (Thermometer DT-610B, CEM).

### Electrophysiology

C57BL/6 mice (male, 4 weeks old) were anesthetized, and the brain was rapidly dissected out. Acute coronal brain slices were prepared in an ice-cold cutting buffer (in mM: 234 sucrose, 2.5 KCl, 1.25 NaH_2_PO_4_, 24 NaHCO_3_, 11 glucose, 0.5 CaCl_2_, 10 MgSO_4_, saturated with 95% O_2_ and 5% CO_2_) with the 300-μm thickness on a vibratome (Leica, VT1000S). Subsequently, the brain slices were recovered in a recovery artificial cerebrospinal fluid (aCSF) containing (in mM) 124 NaCl, 3 KCl, 1.25 NaH_2_PO_4_, 26 NaHCO_3_, 10 glucose, 6.5 MgSO_4_, 1 CaCl_2_, saturated with 95% O_2_ and 5% CO_2_ at 35 °C, and maintained at an ambient temperature thereafter. The brain slices containing the hypothalamic region were used for electrophysiology experiments in a recording aCSF solution containing (in mM) 124 NaCl, 3 KCl, 1.25 NaH_2_PO_4_, 26 NaHCO_3_, 10 glucose, 1.3 MgSO_4_, 2.5 CaCl_2_, saturated with 95% O_2_ and 5% CO_2_ at 37 °C. Hypothalamic ARC neurons were whole-cell patched under a current-clamp configuration with the internal solution containing (in mM)130 K-gluconate, 10 KCl, 10 HEPES, 0.2 EGTA, 4 ATP-Mg, 0.5 GTP-Na2, 10 phosphocreatine-Na_2_ (pH = 7.25 and osmolality = 290 mOsm), and a change in the spontaneous firing frequency was monitored before and after the application of DMSO or SHQA (4.3 ng/µl).

### Biochemical assays

For the glucose tolerance test, we intraperitoneally injected 10 ml glucose per kg of body weight into experimental mice after 8 h of fasting. The blood glucose was measured from mice’s tails at 0, 15, 30, 60, and 120 min after the glucose injection. The plasma triglyceride and total cholesterol were measured using pharmaceutical enzymatic kits according to the manufacturer's instructions (Asan Pharm, Seoul, South Korea). Free fatty acids in plasma were assessed with acyl-CoA synthetase–acyl-CoA oxidase (ACS-ACOD) with the NEFA-HR (non-esterified fatty acids) reagent according to the manufacturer's instruction (Wako, Tokyo, Japan).

### Total RNA isolation, cDNA synthesis, and quantitative real-time PCR

The total RNA was extracted from the brown adipose tissue, white adipose tissue, and hypothalamus using Trizol reagent (Invitrogen Life Technologies, USA) according to the manufacturer’s instructions. We added TRIzol reagent (1.0 mL) to each 50 mg of the hypothalamus for homogenizing the brains. 0.2 mL of the chloroform was added to the homogenized lysate of the brain with TRIzol reagent then it was incubated at room temperature. The supernatant was collected after centrifugation. We added 0.5 mL of isopropanol to this supernatant for the aqueous phase isolation. It was incubated at room temperature and centrifuged at 12,000 rpm (4 °C) for 10 min. Total RNAs are precipitated in a white gel-like pellet so we collected this white gel-like pellet. 70% ethanol was added to this pellet and centrifuged at 12,000 rpm (4 °C) for 5 min for washing. The pellet, total RNA, was dissolved in diethylpyrocarbonate (DEPC)-treated water, and the concentration of total RNA was measured using the NanoDrop (Thermo Scientific). For cDNA synthesis, SuperScript^®^III First-Strand Synthesis System for RT-PCR kit (Invitrogen Life Technologies, USA) was used to reverse-transcribe total RNA (5 μg). cDNA expression was quantified by real-time PCR using the PowerSYBR^®^Green PCR Master Mix (Appliedbiosystems) on the QuantStudio™ 3 Real-Time PCR System (Appliedbiosystems, USA), and primers with the following sequences: *Ucp1*, 5′- AGGCTTCCAGTACCATTAGGT-3′ and 5′-CTGAGTGAGGCAAAGCTGATTT-3′; *Pgc1a*, 5′-TATGGAGTGACATAGAGTGTGCT-3′ and 5′-CCACTTCAATCCACCCAGAAAG-3′; *Prdm16*, 5′-CCAAGGCAAGGGCGAAGAA-3′ and 5′-AGTCTGGTGGGATTGGAATGT-3′; *Ppara*, 5′-AGAGCCCCATCTGTCCTCTC-3′ and 5′-ACTGGTAGTCTGCAAAACCAAA-3′; *Pparg*, 5′-TCGCTGATGCACTGCCTATG-3′ and 5′-GAGAGGTCCACAGAGCTGATT-3′; *Ucp2*, 5′-ATGGTTGGTTTCAAGGCCACA-3′ and 5′-CGGTATCCAGAGGGAAAGTGAT-3′; *Cpt1*, 5′-GCACACCAGGCAGTAGCTTT-3′ and 5′-CAGGAGTTGATTCCAGACAGGTA-3′; *Tfam*, 5′-ATTCCGAAGTGTTTTTCCAGCA-3′ and 5′-TCTGAAAGTTTTGCATCTGGGT-3′; *Acc*, 5′-CCTTTGGCAACAAGCAAGGTA-3′ and 5′-AGTCGTACACATAGGTGGTCC-3′; *lepRb*, 5′-TGGTCCCAGCAGCTATGGT-3′ and 5′-ACCCAGAGAAGTTAGCACTGT-3′; *lepRb2*, 5′-GTCTTCGGGGATGTGAATGTC-3′ and 5′-ACCTAAGGGTGGATCGGGTTT-3′; *Crhr1*, 5′-GGAACCTCATCTCGGCTTTCA-3′ and 5′-GTTACGTGGAAGTAGTTGTAGGC-3′; *Thr*, 5′-GAACAGCTCAAGAATGGTGGC-3′ and 5′-GAATCGAACTCTGCACTTCTCTC-3′; *Hcrtr1*, 5′-GAGGATTCCCTCTCTCGTCG-3′ and 5′-GGTGTAGGTATTCCCTCCACA-3′; *Hcrtr2*, 5′-GAGGATTCCCTCTCTCGTCG-3′ and 5′-GGTGTAGGTATTCCCTCCACA-3′; *Ptpn2* *Bdnf*, 5′-TCATACTTCGGTTGCATGAAGG-3′ and 5′-AGACCTCTCGAACCTGCCC-3′; *Gabra1*, 5′-AAAAGTCGGGGTCTCTCTGAC-3′ and 5′-CAGTCGGTCCAAAATTCTTGTGA-3′; *Gabra2*, 5′-GGACCCAGTCAGGTTGGTG-3′ and 5′-TCCTGGTCTAAGCCGATTATCAT-3′; *Gabrb1*, 5′-TCCCGTGATGGTTGCTATGG-3′ and 5′-CCGCAAGCGAATGTCATATCC-3′; *Gabrb2*, 5′-ATGTCGCTGGTTAAAGAGACG-3′ and 5′-CTGCCACTCGGTTGTCCAAA-3′; *Gabrg1*, 5′-GAAGCTGAAAAACAAGACTTCGG-3′ and 5′-ATGCTGTTCATGGGAATGAGAG-3′; *Gabrg2*, 5′-AGAAAAACCCTCTTCTTCGGATG-3′ and 5′-GTGGCATTGTTCATTTGAATGGT-3′; *Vgat*, 5′-ACCTCCGTGTCCAACAAGTC-3′ and 5′-CAAAGTCGAGATCGTCGCAGT-3′; *Cd137*, 5′-CCTGTGATAACTGTCAGCCTG-3′ and 5′-TCTTGAACCTGAAATAGCCTG-3′; *Tbx1*, 5′-CTGTGGGACGAGTTCAATCAG-3′ and 5′-TTGTCATCTACGGGCACAAAG-3′; *Tmem26*, 5′- TTCCTGTTGCATTCCCTGGTC-3′ and 5′- GCC GGA GAA AGC CAT TTG T-3′; *Cart*, 5’-GCCAAGTCCCCATGTGTGAC-3’ and 5’-GCCAAGGCTGTGGGCAAGGT-3’; *Actb*, 5'-GGCTGTATTCCCCTCCATCG-3' and 5'-CCAGTTGGTAACAATGCCATGT-3'. The cycle threshold (Ct) value, which was obtained by monitoring the fluorescence signal for each cycle, was calibrated using the β-actin (Actb). Gene expressions were calculated by the 2-ΔΔCT method.

### Western blot analysis

The white and brown adipose tissue of the mice were homogenized on ice using RIPA buffer (Sigma). The homogenates were centrifuged at 15,000 rpm for 10 min, then the supernatants were used for the determination of protein concentrations by BCA protein assay (#23,225, Thermo Fisher, USA). 30 µg of proteins were used for separation by 10% polyacrylamide gel electrophoresis and transferred to PVDF membranes (Millipore). After blocking in 3% bovine serum albumin, the membrane was incubated with rabbit anti-UCP1 (1:1000, Ab100983, Abcam), rabbit anti-PCG1α (1:1000, Novus, NBP1-04676), and rabbit anti- β-tubulin (1:1000, Cell Signaling Technology, #2146) overnight at 4 °C. After the membranes were washed, they were incubated for one hour with HRP conjugated Donkey anti-Rabbit IgG antibody (1: 10,000, Bethyl, A120-108P). The full-length images were provided in Supplementary Figs. 1 and 2. The bands were visualized using Alliance Q9 mini (UVITEC, Cambridge, UK) with ECL reagent (Bio-Rad), and the intensity was quantified using Image J software (National Institutes of Health).

### Statistical analysis

Experimental values were shown as mean ± standard error of the mean (S.E.M.) and evaluated with one-way ANOVA by Tukey’s post hoc test. The statistical analysis was performed using the GraphPad PRISM software (GraphPad Prism Software Inc., version 8, CA, USA). *P* values of < 0.05 were deemed significant.

## Supplementary Information

Supplementary Information.
