# Supplementary material for: Hypothalamic administration of sargahydroquinoic acid elevates peripheral thermogenic signaling and ameliorates high fat diet-induced obesity through the sympathetic nervous system
Source: Sci Rep. 2021 Oct 29;11:21315. doi: 10.1038/s41598-021-00074-3 (PMC8556287; doi:10.1038/s41598-021-00074-3)
Supplement: Supplementary file 1 — Supplementary Information. [file 41598_2021_74_MOESM1_ESM.pdf]

BAT

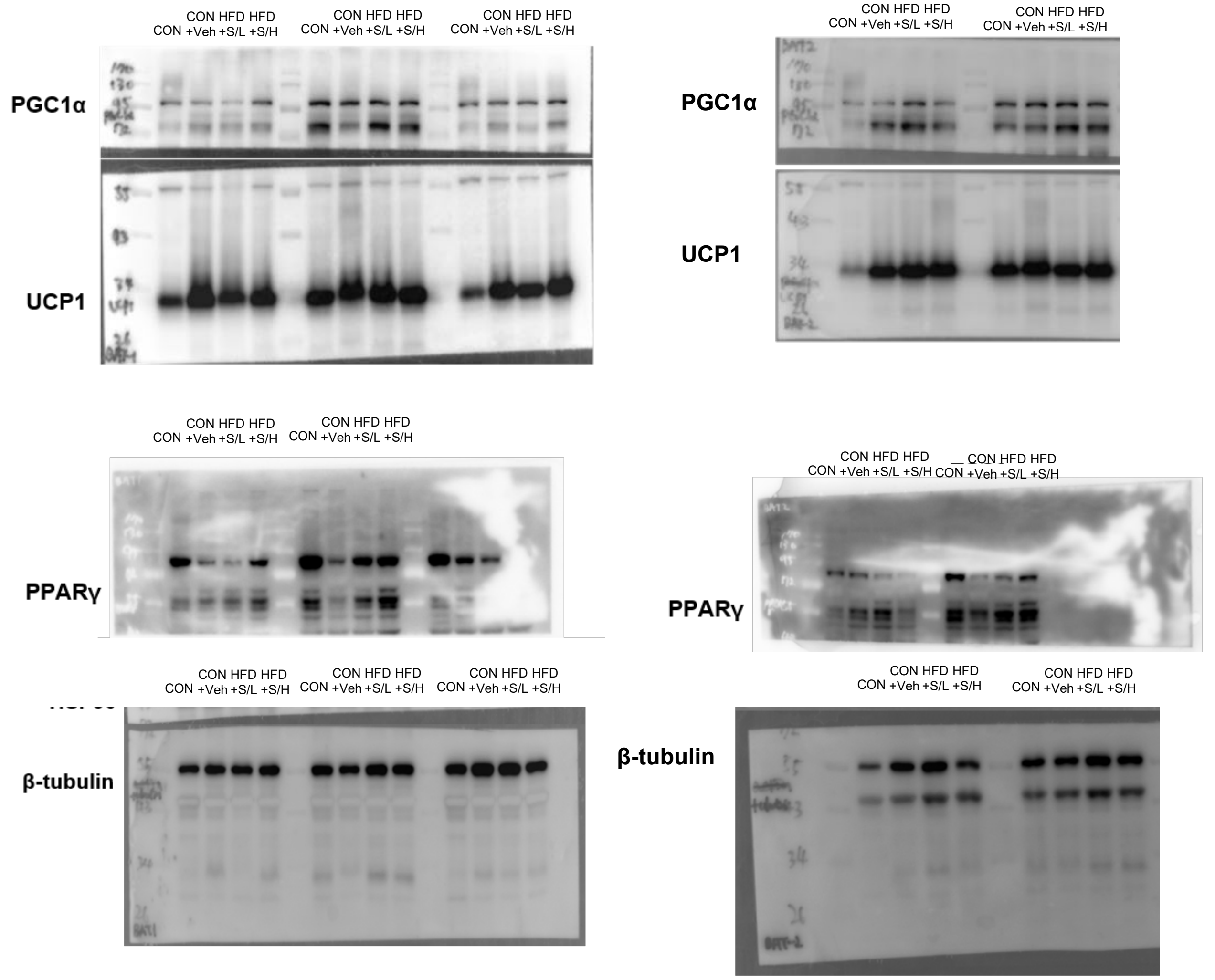

Supplementary figure 1. The images of full-length blots of UCP1, PGC1α, PPARγ and β-tubulin proteins in BAT

Standard C57BL/6 mice (4 weeks old) were fed with chow diet (CON) and high-fat diet (HFD) with the hypothalamic injection of PBS (CON or HFD+Veh, three times a week), low dose SHQA (HFD+SHQA/L, 20 ng/µl, three times a week) and high dose (HFD+SHQA/H, 100 ng/µl, three times a week) for 12 weeks. The samples derived from the same experiment and that gels/blots were processed in parallel and these images were used for the representative blot pictures in Fig. 5B.

Suppl. Fig. 2

WAT

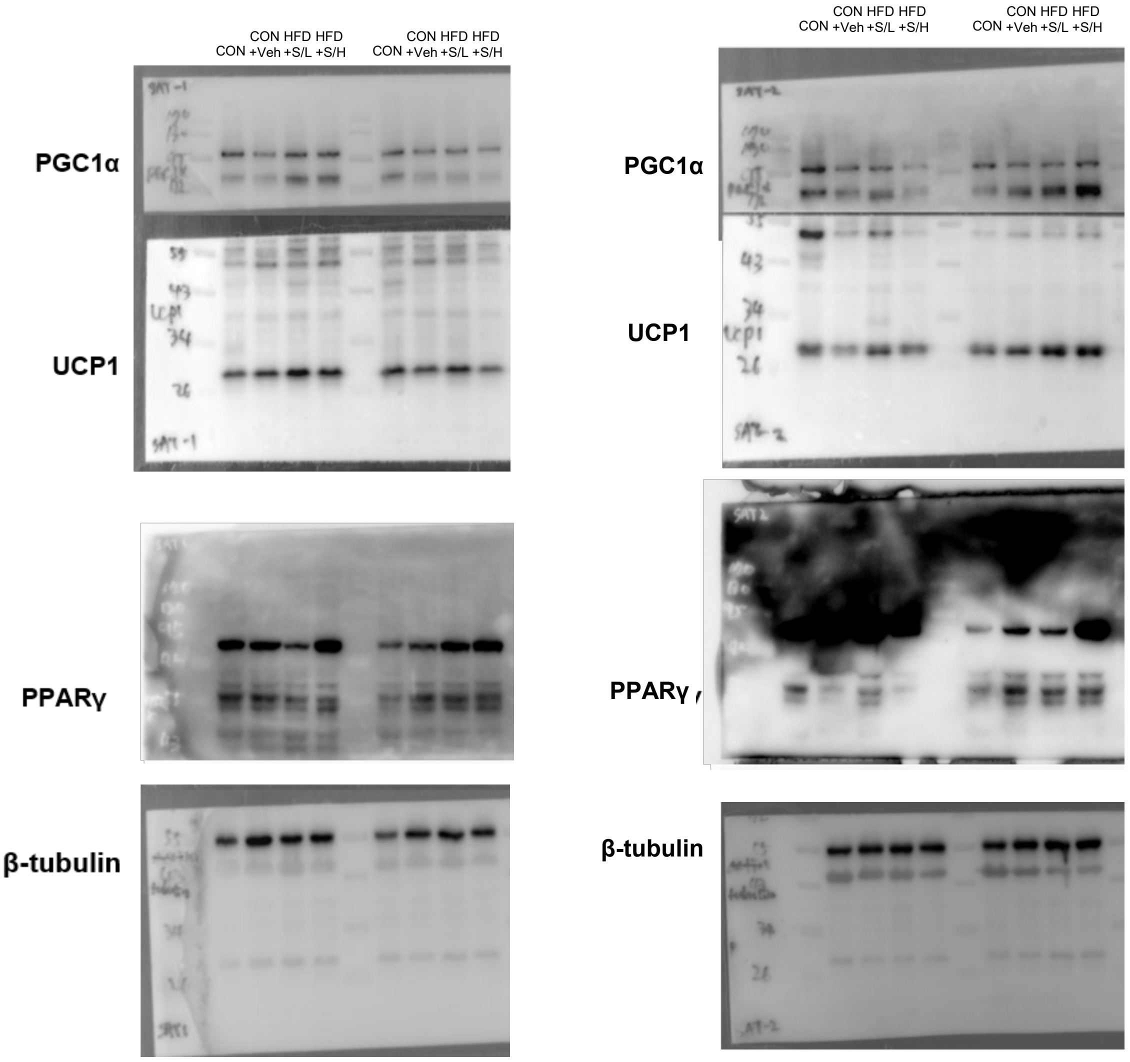

Supplementary figure 2. The images of full-length blots of UCP1, PGC1α, PPARγ and β-tubulin proteins in WAT

Standard C57BL/6 mice (4 weeks old) were fed with chow diet (CON) and high-fat diet (HFD) with the hypothalamic injection of PBS (CON or HFD+Veh, three times a week), low dose SHQA (HFD+SHQA/L, 20 ng/μl, three times a week) and high dose (HFD+SHQA/H, 100 ng/μl, three times a week) for 12 weeks. The samples derived from the same experiment and that gels/blots were processed in parallel and these images were used for the representative blot pictures in Fig. 6B.
